# Modulating and monitoring the functionality of corticostriatal circuits using an electrostimulable microfluidic device

**DOI:** 10.1186/s13041-023-01007-z

**Published:** 2023-01-20

**Authors:** Sukmin Han, Seokyoung Bang, Hong Nam Kim, Nakwon Choi, Sung Hyun Kim

**Affiliations:** 1 Department of Neuroscience, Graduate School, Kyung Hee University, Seoul, 02447 Republic of Korea; 2 Brain Science Institute, Korea Institute of Science and Technology (KIST), Seoul, 02792 Republic of Korea; 3 Department of Physiology, School of Medicine, Kyung Hee University, Seoul, 02447 Republic of Korea; 4 Medical Research Center for Bioreaction to Reactive Oxygen Species and Biomedical Science Institute, School of Medicine, Kyung Hee University, Seoul, 02447 South Korea

**Keywords:** Microfluidic device, Corticostriatal (CStr) circuit, Synapse, Ca^2+^ dynamics, Action potential, Synaptic transmission

## Abstract

The central nervous system is organized into different neural circuits, each with particular functions and properties. Studying neural circuits is essential to understanding brain function and neuronal diseases. Microfluidic systems are widely used for reconstructing and studying neural circuits but still need improvement to allow modulation and monitoring of the physiological properties of circuits. In this study, we constructed an improved microfluidic device that supports the electrical modulation of neural circuits and proper reassembly. We demonstrated that our microfluidic device provides a platform for electrically modulating and monitoring the physiological function of neural circuits with genetic indicators for synaptic functionality in corticostriatal (CStr) circuits. In particular, our microfluidic device measures activity-driven Ca^2+^ dynamics using Ca^2+^ indicators (synaptophysin-GCaMP6f and Fluo5F-AM), as well as activity-driven synaptic transmission and retrieval using vGlut-pHluorin. Overall, our findings indicate that the improved microfluidic platform described here is an invaluable tool for studying the physiological properties of specific neural circuits.

**Supplementary Information:**

The online version contains supplementary material available at 10.1186/s13041-023-01007-z.

## Introduction

Neurons in the brain are organized into many neural circuits—each connected by specific types of neurons—that span various brain regions. Understanding neural circuits is essential to understanding the functional connections among brain regions, which, in turn, is crucial for identifying mechanisms related to circuit dysfunctions in brain diseases [1–3]. There is rapid and growing interest in investigating how neural circuits are structurally formed and functionally operate [4, 5]. A system capable of modulating and monitoring a neural circuit in an intact, in vitro three-dimensional structure would be ideal to achieve this goal, but available technologies are not suited to the complexity and diversity of the brain.

Research interest in reconstructing neural circuits or networks using in vitro systems has been growing continuously. Microfluidic systems in particular are among the most practical techniques for in vitro reconstruction of neural circuits, providing a structural platform for investigating the formation of numerous types of circuits down to the single-circuit level. Since their first use for the reconstruction of neural circuits about two decades ago, microfluidic systems have undergone rapid development and have been applied in various forms to the study of neural circuits [6–10]. The in vitro circuit platforms that have grown out of these efforts have provided controllable environments for measuring the dynamics and functionality of neural circuits [11]. They have also proven very useful for studying synapses in the setting of a specific circuit [9, 12]. However, studying the functional physiology of a neural circuit using a microfluidic system is still plagued by limitations of technical difficulty and high cost, highlighting the need for technical improvements.

Corticostriatal (CStr) circuits are critical components of forebrain functions, including motivation, cognition, reward and motor control, and they are disrupted in several neurological disorders [13–16]. There are a number of neural circuits in which the cortex, basal ganglia, and thalamus are interconnected in large-scale loops that are widely involved in motivated behavior [13, 15]. Cortical output neurons drive striatal activity through the release of glutamate. Striatal outputs, in turn, release γ-amino butyric acid (GABA) and exert inhibitory control over downstream basal ganglia targets [17, 18]. A previous study demonstrated that alteration of glutamate transmission in cortical synapses influences GABA output onto its corresponding striatal synapses [19]. Furthermore, dysfunction of synapses in CStr circuits contributes to neurological and neuropsychiatric disorders, such as autism spectrum disorder (ASD), Huntington’s disease (HD), and schizophrenia [20–22]. Thus, it is very important to understand how synapses in CStr circuits function and how dysfunction of these circuits results in neurological diseases.

In the present study, we developed an improved microfluidic device capable of electrically modulating and monitoring the physiological properties of CStr circuits. The electrode hole in this improved microfluidic device readily accommodates an electrode probe, which can be quickly and easily connected. By combining a genetic Ca^2+^ indicator (synaptophysin-GCaMP6f) and pHluorin-based assay system (vGlut1-pHluorin), we successfully modulated and monitored important physiological parameters using our advanced microfluidic device, including activity-driven Ca^2+^ influx at synapses of CStr circuits and synaptic transmission and synaptic retrieval of CStr circuits.

## Materials and methods

### Microfluidic device

A master mold was made with SU-8 photoresist on a silicon wafer using a dual-thicknesses photolithography process. The thicker layer (100 μm) is for the cell-plating chamber and reservoir, and the thinner layer (3 μm) is for microchannels. The microfluidic device for cell culture was made of a PDMS mixture consisting of Sylgard 184 (Dow Corning, Michigan, USA) and its curing agent (ratio 10:1). The PDMS mixture was poured into the master mold, and after degassing for 30 min and baking for 1 h at 80 °C, the PDMS replica was detached from the master mold. A plasma coating was applied to the detached PDMS replica and cover glass using a plasma system (80 W, 50 kHz, 6.50 × 10–1 torr, 40 s; Femtoscience, South Korea), and the PDMS replica was mounted on the coverslip. Microfluidic devices were placed under UV light for 15 min, then coated with poly-L-ornithine overnight and washed with phosphate-buffered saline (PBS) before cell plating.

### Primary neuron culture and electroporation

Primary prefrontal cortical and striatal neurons were isolated from postnatal (1–2 d old) Sprague–Dawley rats (DBL; Strain code: NTac:SD) and plated on the poly-L-ornithine-coated microfluidic device. Immediately before plating, cortical neurons were electroporated with vGlut1-pHluorin (vG-pH), vGlut1-pHluorin-mCherry (vG-pH-mCh), synaptophysin-pH (Physin-pH), synapto-pHluorin (Syn-pH), or synatophysin-GCaMP6f (Physin-GC6f) plasmids using a NEPA21 Super Electroporator (Nepa Gene). Transfected cortical neurons were plated first on one side and striatal neurons were subsequently plated on the other side. Neurons were further incubated and culture media were refreshed every other day. Animal treatments in this study were carried out in accordance with Animal Care and Use Guidelines, and all experiments were approved by the Animal Care Committee of Kyung Hee University.

### Optical setup for imaging physiological parameters

Live-cell imaging was performed on DIV14-21 neurons electroporated with vG-pH, vG-pH-mCh, Syn-pH, Physin-pH or Physin-GC6f before plating on the microfluidic device. Microfluidic devices with coverslip were mounted on the stage of a custom-built, laser-illuminated epifluorescence microscope (Zeiss Observer). Live-cell images were acquired with an Andor iXon Ultra 897 (Model #DU-897U-CS0-#BV) back-illuminated EMCCD camera. A diode-pumped OBIS 488 and 561 laser (Coherent), shuttered by synchronizing the TTL on/off signal from the EMCCD camera during acquisition, was utilized as a light source. Fluorescence excitation/emission and collection were achieved using a 40 × Fluar Zeiss objective lens (1.3 NA) and 500–550 nm emission and 498 nm dichroic filters (Chroma). Action potentials (APs) were evoked by passing a 1 ms current pulse (~ 7 mA), yielding fields of about 1 V/cm via platinum-iridium electrodes using an A310 Accupulser and A385 stimulus isolator (World Precision Instruments). Neurons were perfused with Tyrode’s buffer consisting of 119 mM NaCl, 2.5 mM KCl, 2 mM CaCl_2_, 2 mM MgCl_2_, 25 mM HEPES, 30 mM glucose, 10 μM 6-cyano-7-nitroquinoxaline-2,3-dione (CNQX), and 50 μM D,L-2-amino-5-phosphonovaleric acid (AP5), adjusted to pH 7.4. All experiments were carried out at 30 °C. pHluorin Images were acquired at 2 Hz with a 50-ms exposure, and Ca^2+^ images were acquired at 100 Hz with a 0.973-ms exposure.

### Immunofluorescence

For immunofluorescence analysis, DIV14-21 neurons in the microfluidic device were fixed with 4% paraformaldehyde (PFA) for 30 min at room temperature (RT). The fixation buffer was rinsed out with PBS, after which neurons were permeabilized with 0.2% Triton X-100 for 30 min and subsequently incubated for 30 min with a blocking solution containing 5% bovine serum albumin (BSA). Anti-Tau (Merck Millipore), anti-MAP2 (Merck Millipore), anti-synapsin (Synaptic Systems), and anti-Shank3 (Synaptic Systems) antibodies were applied to microchannels and the microfluidic device was incubated overnight at 4 °C. After incubating with primary antibodies, neurons were washed with PBS for 30 min and subsequently incubated with Alexa 488- and/or Alexa 546-conjugated secondary antibodies (Invitrogen) for 1 h, providing different color combinations as needed.

### Image analysis

Images were analyzed with ImageJ (http://rsb.info.nih.gov/ij) using a custom-written plugin (http://rsb.info.nih.gov/ij/plugins/time-series.html). Synaptic boutons were selected as oval regions of interest (diameter, 10 pixels), and the intensity of fluorescence at synapses was measured. Fluorescence intensity values obtained in pHluorin-based assays and Ca^2+^ dynamics were analyzed with Origin Pro (ver. 2020). The kinetics of endocytosis was fitted using a single-exponential decay function.

## Results

### Design and construction of an microfluidic device for investigating the physiology of neural circuits

**Fig. 1:**
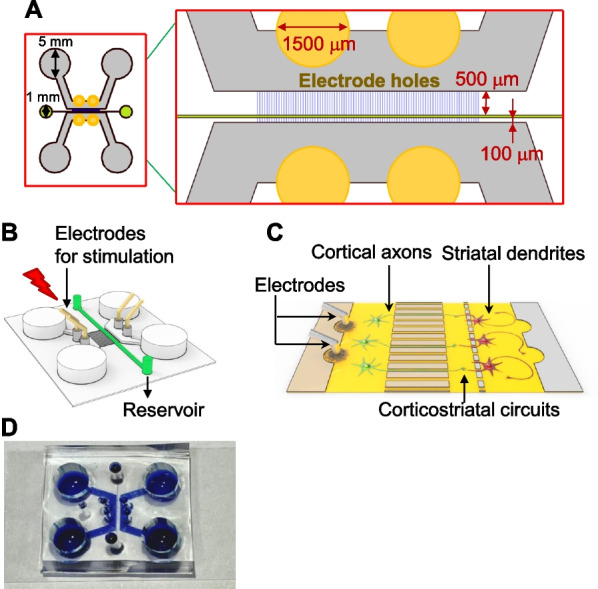
Schematic diagram of an electrostimulable microfluidic device system. **A** Schematic diagram of the microfluidic device system (left). Magnified view of the microchannel area with electrode holes (right). Yellow circles indicate electrode holes for simple insertion of electrodes. Microchannel (blue) and synapse canal (green) are localized between cortical and striatal areas. **B** 3D schematic diagram of the microfluidic device. Electrodes for electrical stimulation are connected via electrode holes (yellow). The reservoir in the synapse canal is directly connected to the synaptic canal to allow for perfusion. **C** Schematic depiction of the microfluidic device with CStr circuits. Cortical axons pass through axonal microchannels, and striatal dendrites pass through dendrite microchannels. **D** The microfluidic device with attached cover glass after plasma coating

The investigation of neural circuit networks is among the most important current research themes in neuroscience. A number of studies have used microfluidic devices for studying specific neural circuits, an approach that is rapidly growing in popularity [8, 9, 23]. Here, we designed and built a microfluidic device for monitoring specific neural circuits—the CStr circuit in the current application—by modulating their activity. Our device was designed with an appropriate channel length and width to achieve this goal. Furthermore, electrode holes were added to the device to enable facile, accurate electrical stimulation (Fig. 1A, B). This microfluidic device provides compartmentalized cell culture platforms that allow isolation of two types of neurons (cortical and striatal in the current configuration) for reconstruction of specific brain circuits (Fig. 1C). Our microfluidic device is composed of three compartments: (1) a neuronal chamber, the largest area (height, 100 μm) in which neurons are placed; (2) a thinner (height, 3 μm) neurite microchannel, accommodating both axon and dendrite outgrowth from each side; and (3) a synapse canal, where synapse connections from each side are made. Additionally, and most importantly, we placed electrode holes in the area juxtaposed to the neurite microchannel. To construct this microfluidic device, we utilized two layers of photoresist (SU-8) for the template and polydimethylsiloxane (PDMS), a type of silicon rubber, for the chamber body. PDMS is widely used because it offers several advantages for use in stamps or molds, including (i) low interfacial free energy for ease of demolding and material transfer; (ii) stability against chemicals, heat, and humidity; and (iii) optical transparency for UV-assisted crosslinking of materials (Fig. 1D) [24].

### Successful reconstitution of CStr circuits in the microfluidic device platform

**Fig. 2:**
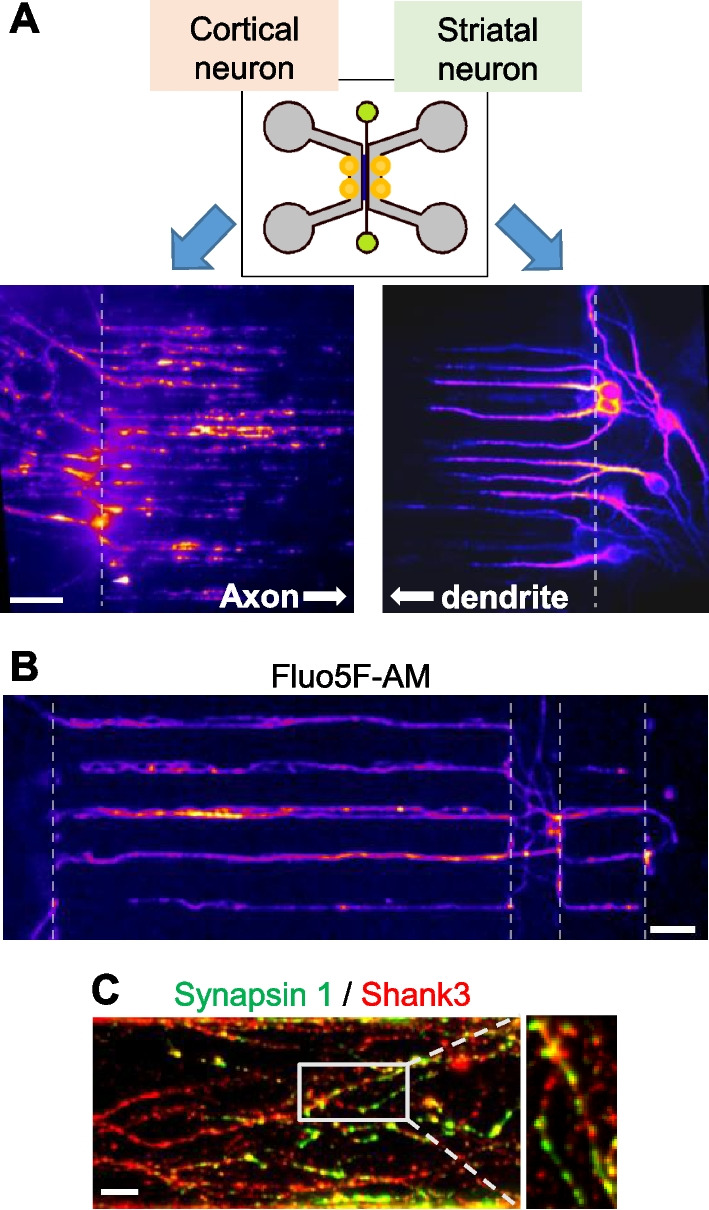
Successful formation of CStr circuits in the microfluidic device system. **A** Axons in cortical neurons (left) and dendrites in striatal neurons (right) successfully extended to each microchannel. Axons in the cortical neuron area and dendrites in the striatal neuron area, identified by immunostaining for the axonal marker Tau and dendritic marker MAP2, respectively. Scale bar: 20 μm. Merged image shows synapse formation between cortical axons and striatal dendrites. Scale bar: 50 μm. **B** Visualization of all connections in CStr circuits by incubation with Fluo5F-AM. Scale bar: 50 μm. **C** Representative image of synapse formation in the synapse canal. CStr circuits in the microfluidic device were immunostained for the presynaptic marker synapsin I (green) and postsynaptic marker Shank3 (red). Right, magnified imaged of boxed region. Scale bar: 10 μm

To verify that our microfluidic device provides a structural neural circuit platform for stably constructing CStr circuits, we placed each type of cell—cortical neurons and striatal neurons—in the corresponding areas on each side of the neuronal chamber. Neurons in the microfluidic device were further incubated for at least 14 d in vitro (DIV14). During incubation, both axons and dendrites of each type of neuron elongated through the microchannels from each side of the neuronal chamber. To examine the growth of cortical axons or striatal dendrites in microchannels and synapse formation in the synapse canal, we performed an immunofluorescence (IF) analysis of axonal and dendrite markers using anti-tau and anti-MAP2 (microtubule-associated protein 2) antibodies, respectively. As shown in Fig. 2A, cortico-axons and striatal-dendrites successfully expanded from their corresponding microchannels, resulting in well-formed synapses in the synapse canal. Furthermore, the Ca^2+^-indicator dye, Fluo5F-AM, clearly revealed formation of entire circuits (Fig. 2B, C). Collectively, these findings demonstrate that our microfluidic device provides an effective structural platform for neural circuit formation.

### Monitoring Ca^2+^ dynamics in CStr circuits during circuit activity using the microfluidic chamber

**Fig. 3:**
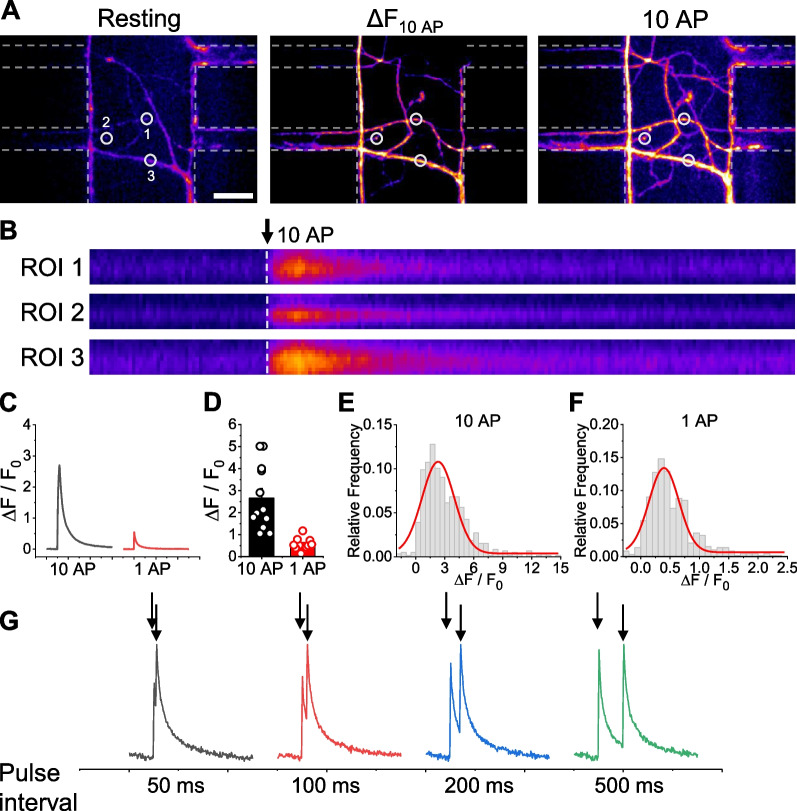
Monitoring of activity-driven Ca^2+^ dynamics in CStr circuits in the electrostimulable microfluidic device using Fluo5F-AM. **A** Representative Fluo5F-AM images of Ca^2+^ dynamics in response to neural activity (10 AP stimulus) at synapses of CStr circuits. Left, resting; middle, ∆F10AP; right, peak 10 AP. CStr circuits were incubated with Fluo5F-AM (5 μM) and stimulated with 10 APs at 100 Hz. Scale bar: 20 μm. **B** Kymograph images of Fluo5F-AM showing Ca^2+^ dynamics in response to 10-AP stimulation at the corresponding region of interest (ROI) in boutons in the synapse canal of the microfluidic chip. Arrow indicates initial point of stimulation. **C** Representative ensemble average Fluo5F-AM traces showing Ca^2+^ dynamics in response to microfluidic chip stimulation with 10 APs or 1 AP. **D** Ca^2+^ influx in arbitrary units (a.u.) in response to stimulation of the microfluidic chip with 10 APs or 1 AP, expressed as means ± SEM. [Ca^2+^]_10AP_ = 2.66 ± 0.43 a.u. (n = 12); [Ca^2+^]_1AP_ = 0.55 ± 0.08 a.u. (n = 12). **E**, **F** Distribution of Ca^2+^-influx responses to 10 APs (**E**; n = 586) and 1 AP (**F**; n = 587) in individual boutons. **G** Representative traces of Ca^2+^ dynamics in response to paired-pulse stimulation at different pulse intervals (50, 100, 200, and 500 ms). Arrows indicate the point of stimulation

**Fig. 4:**
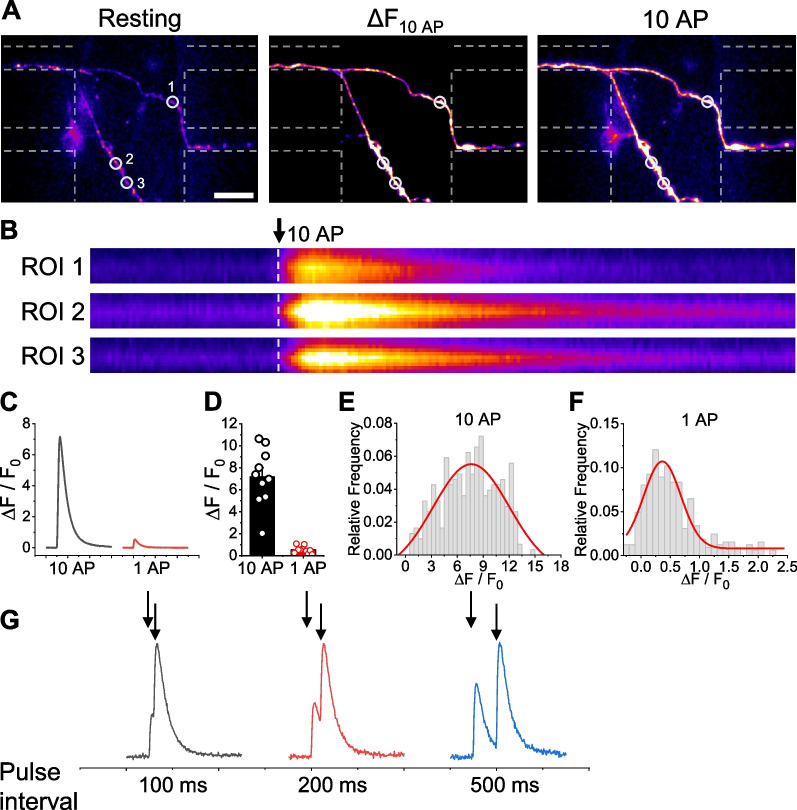
Monitoring of activity-driven Ca^2+^ dynamics in CStr circuits in the electrostimulable microfluidic device using Physin-GC6f. **A** Representative Physin-GC6f images of Ca^2+^ dynamics in response to neural activity (10 AP stimulus) at synapses of CStr circuits. Left, resting; middle, ∆F10AP; right, peak 10 AP. Scale bar: 20 μm. **B** Kymograph images of Physin-GC6f showing Ca^2+^ dynamics at the corresponding ROI in boutons in the synapse canal of the microfluidic chip in response to stimulation with 10 APs. Arrow indicates point of stimulation. **C** Representative ensemble Physin-GC6f traces showing Ca^2+^ dynamics in response to microfluidic chip stimulation with 10 APs or 1 AP. **D** Ca^2+^ influx, in arbitrary units (a.u.), in response to microfluidic chip stimulation with 10 APs or 1 AP, expressed as means ± SEM. [Ca^2+^]_10AP_ = 7.17 ± 0.82 a.u. (n = 10); [Ca^2+^]_1AP_ = 0.54 ± 0.10 a.u. (n = 10). **E**, **F** Distribution of Ca^2+^-influx responses to 10 APs (**E**; n = 305) and 1 AP (**F**; n = 324) in individual boutons. **G** Representative traces of Ca^2+^ dynamics in response to paired-pulse stimulation at different pulse intervals (100, 200 and 500 ms). Arrows indicate the point of stimulation

Having confirmed successful construction of a structural platform for reconstructing CStr circuits, we next sought to demonstrate that our microfluidics system can provide physiological monitoring of circuit responses to electrostimulation. Because Ca^2+^ signaling is essential for neural activity, we monitored Ca^2+^ dynamics in CStr circuits in our microfluidic device using two different Ca^2+^ indicators. First, we employed the chemical Ca^2+^ indicator, Fluo5F-AM. After treating CStr circuits in the microfluidic chamber with Fluo5F-AM and washing, the microfluidic chip was connected to the electrode and stimulated with 1 or 10 action potentials (APs) at 100 Hz. Synapses of CStr circuits showed strong responses to corresponding electrical activities in the synapse canal of the microfluidic chip (Fig. 3A–F; Additional file 1: Video S1). We further stimulated CStr circuits at various paired-pulse intervals (50, 100, 200, and 500 ms). As shown in Fig. 3G, Ca^2+^ dynamics in CStr circuits clearly exhibited paired-pulse responses. Second, we made use of the synapse-specific genetic Ca^2+^ indicator, synaptophysin-GCaMP6f (Physin-GC6f), which has been employed in a number of studies for monitoring synaptic Ca^2+^ dynamics [25–27]. To this end, cortical neurons were electroporated with Physin-GC6f and plated the resulting transfected cells in the neuronal chamber of the microfluidic device. CStr circuits formed with 14 d of plating (14DIV), and a Physin-GC6f–positive circuit was selected for monitoring of Ca^2+^ dynamics using the same experimental regime as used for Fluo5F-AM. Physin-GC6f–positive synapses in the CStr circuit showed Ca^2+^ responses upon electrical stimulation that were clearly visible under all stimulation conditions (i.e., 1 AP, 10 APs, paired-pulse) (Fig. 4A–G; Additional file 2: Video S2). Taken together, these results indicate that our microfluidic device is capable of monitoring electrical stimulation-induced Ca^2+^ dynamics in the CStr chip.

### Measurement of synaptic transmission during neural activity using the microfluidic chip

**Fig. 5:**
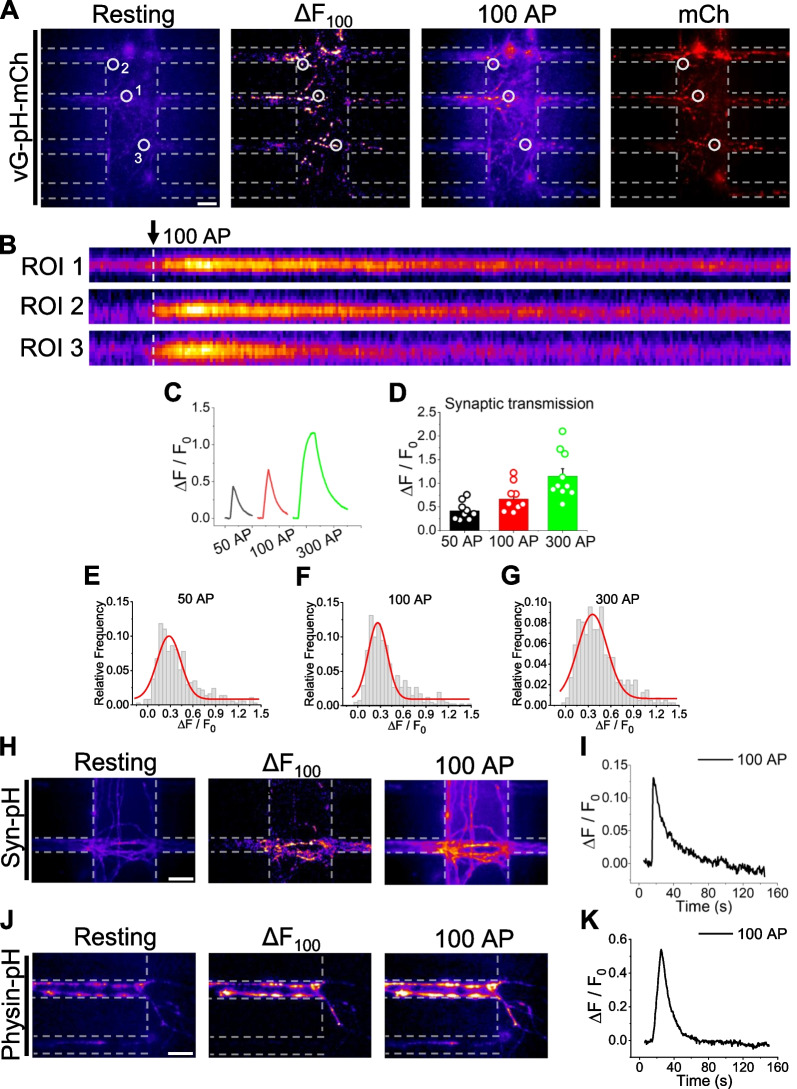
Measurement of synaptic transmission in CStr circuits in the electrostimulable microfluidic device. **A** Representative images of vG-pH-mCh fluorescence in response to neural activity (100 AP stimulation) at synapses of CStr circuits. Left to right: resting, ∆F100AP, peak 100 AP, and mCherry. Scale bar: 20 μm. **B** Kymograph images showing vG-pH-mCh fluorescence at the corresponding ROI of boutons in the synapse canal in response to microfluidic chip stimulation with 100 APs. Arrow indicates point of stimulation. **C** Representative ensemble traces of vG-pH-mCh fluorescence in response to microfluidic chip stimulation with 50, 100 and 300 APs. **D** vG-pH-mCh fluorescence in response to microfluidic chip stimulation with 50, 100, and 300 APs, expressed as means ± SEM. [vG-pH-mCh]_50AP_ = 0.42 ± 0.06 a.u. (n = 9); [vG-pH-mCh]_100AP_ = 0.67 ± 0.09 a.u. (n = 10); [vG-pH-mCh]_300AP_ = 1.15 ± 0.16 a.u. (n = 10). **E–G** Distribution of vG-pH-mCh fluorescence amplitude responses at 50 APs (**E**; n = 372), 100 APs (**F**; n = 411), and 300 APs (**G**; n = 409) in individual boutons. **H** Representative images of Syn-pH fluorescence in response to neural activity (100 AP stimulation) at synapses of CStr circuits. Left, resting; middle, ∆F100AP; right, peak 100 AP. Scale bar: 20 μm. **I** Representative ensemble traces of Syn-pH fluorescence in response to microfluidic chip stimulation with 100 APs. **J** Representative images of Physin-pH fluorescence in response to neural activity (100 AP stimulation) at synapses of CStr circuits. Left, resting; middle, ∆F100AP; right, peak 100 AP. Scale bar: 10 μm. **K** Representative ensemble traces of Physin-pH fluorescence in response to microfluidic chip stimulation with 100 APs

We next investigated synaptic transmission in CStr circuits in the microfluidic system in response to an electrical stimulation. To measure synaptic transmission, we employed a pHluorin-based assay in which pHluorin conjugated to the luminal region of the synaptic vesicle membrane proteins vGlut1, VAMP2, or synaptophysin is used to directly monitor synaptic vesicle exocytosis at nerve terminals [28–30]. We introduced each of these three pHluorin systems—vGlut1-pHluorin (vG-pH) [or vGlut-pH-mCh (vG-pH-mCh)], VAMP2-pHluorin (Syn-pH), and synaptophysin-pHluorin (Physin-pH), respectively—in a CStr circuit in a microfluidic chip and observed physiological synaptic transmission responses under various electrostimulation regimes (50, 100, and 300 APs). As shown in Fig. 5A–G, CStr circuits in the chip transfected with vG-pH-mCh showed vivid synaptic transmission responses under each electrical stimulation condition that increased with increasing stimulation intensity (Additional file 3: Video S3). Circuits expressing Syn-pH or Physin-pH also exhibited clear synaptic transmission responses upon stimulation (Fig. 5H–K). Collectively, these observations demonstrate successful monitoring of synaptic transmission in CStr circuits in our microfluidic system.

### Monitoring synaptic vesicle retrieval after electrical stimulation using the microfluidic device

**Fig. 6:**
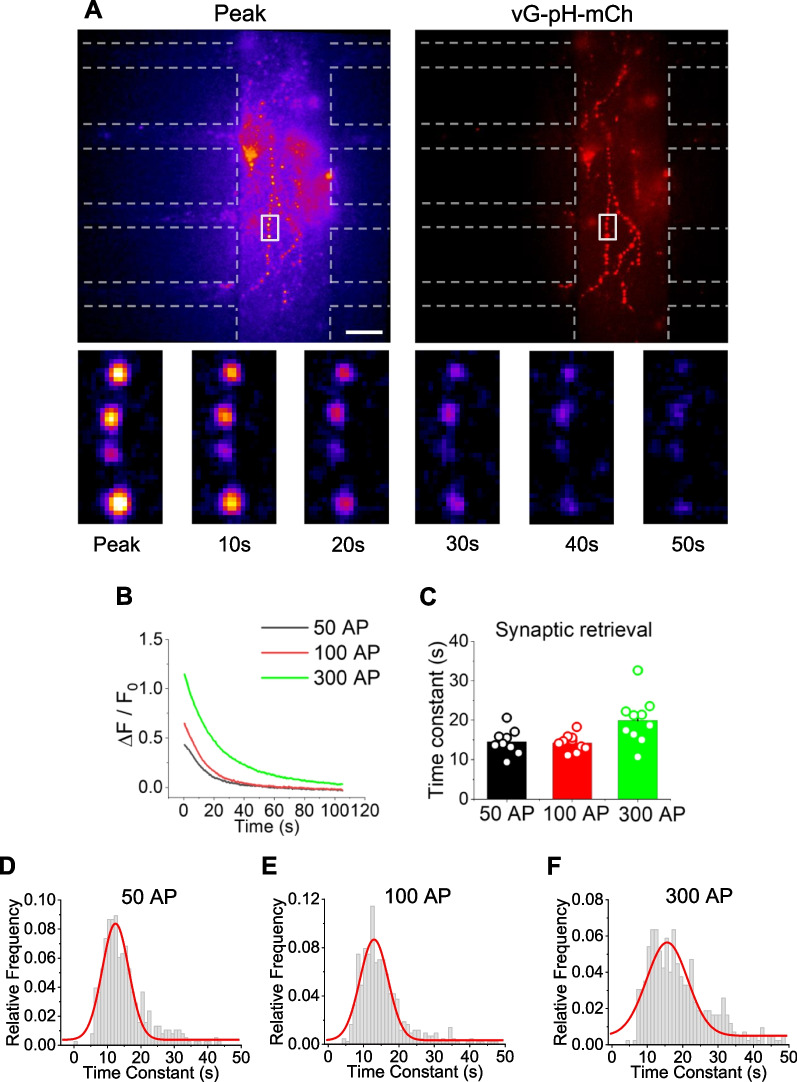
Monitoring of synaptic retrieval in the microfluidic device. **A** Top: Representative snapshot images of vG-pH-mCh fluorescence (left) and corresponding mCherry images (right) in the CStr circuit at the end of microfluidic chip stimulation with 100 APs. Bottom: Time-lapse snapshots of the time course of synaptic vesicle endocytosis. Scale bar: 10 μm. **B** Representative ensemble traces of vG-pH-mCh fluorescence during endocytosis in response to different stimulation conditions (50, 100, and 300 APs). **C** Endocytosis time constants of vG-pH-mCh fluorescence in response to microfluidic chip stimulation with 50, 100, and 300 APs, expressed as means ± SEM. [τ_endo_]_50AP_ = 14.54 ± 1.08 s (n = 9); [τ_endo_]_100AP_ = 14.24 ± 0.67 s (n = 10); [τ_endo_]_300AP_ = 19.92 ± 1.87 s (n = 10). **D–F** Distribution of vG-pH-mCh endocytosis time constants at 50 APs (**D**; n = 381), 100 APs (**E**; n = 429), and 300 APs (**F**; n = 425) in individual boutons

Synaptic retrieval, or synaptic vesicle endocytosis, is an essential process following synaptic vesicle fusion that serves to maintain the functionality of neural circuits for subsequent rounds of stimulation. To test whether synaptic retrieval of CStr circuits can be monitored and analyzed in our microfluidic system, we also used the pHluorin based system, focusing on the response after electrical stimulation, which represents the course of synaptic vesicle endocytosis. To this end, we analyzed the decay of pHluorin fluorescence after stimulation of CStr circuits with 50, 100, or 300 APs. Endocytosis of synaptic vesicles was detected under each of the various stimulation conditions (Fig. 6A, B). An analysis of the time constant of synaptic vesicle endocytosis (τ_endo_) showed that the mean value of τ_endo_ was ~ 14 s under 50 and 100 AP conditions, and slowed to ~ 20 s at 300 APs, values similar to those previously reported [29, 30] (Fig. 6C–F). These findings demonstrate that our microfluidic system is capable of monitoring synaptic vesicle retrieval in the CStr circuit under various stimulation conditions.

## Discussion

The microfluidic device platform—one of the most useful technologies for investigating neural circuits in vitro—has undergone continuous development and improvement, bringing enhanced efficiency to studies of neural circuits. Microfluidic devices for structural studies of neural circuits are currently well established, but these devices lack an efficient way to measure the physiological properties of neural circuits. To stimulate circuit activity, many groups have applied chemicals, such as high levels of KCl, that induce APs or change ion channel activity [22, 31]. Other groups have utilized a coverslip-mounted multiple microelectrode array system or a microelectrode-embedded microfluidic system [23, 32]. These microfluidic systems have enabled researchers to gain a deeper understanding of neural circuits, but they still have limitations. For example, the chemical stimulation method lacks the ability to modulate APs accurately, and microelectrode array systems are relatively expensive and compatible only with a limited range of experiments.

In the present study, we designed and constructed an electrostimulable microfluidic device that is quick and easy to use and tested it using CStr circuits. Two features of our microfluidic device stand out. First, it features two electrode holes at the center of the neuronal chamber adjacent to the microchannel, which provides a convenient connection between the neural circuit and the electrode. Second, it contains a physically divided neuronal chamber with two sub-chambers—one for cortical neurons and the other for striatal neurons—that are connected to axonal and dendritic microchannels and meet in the synapse canal.

Applying our microfluidic system to studies of the CStr circuit, we demonstrated several key parameters. Physiologically, we verified several physiological functionalities. First, we demonstrated the ability to monitor activity-driven Ca^2+^ dynamics using Fluo5F-AM and Physin-GC6f, showing that synapses in CStr circuits exhibited vivid Ca^2+^-influx responses upon modulation of neuronal activity with various stimulation paradigms (Figs. 3, 4). We also monitored synaptic transmission and synaptic retrieval using the pHluorin system (i.e., vG-pH, Syn-pH, and Physin-pH), showing that synapses of CStr circuits expressing pHluorin-conjugated synaptic vesicle membrane proteins exhibited clear synaptic transmission and synaptic retrieval in response to various stimulation regimes (Figs. 5, 6).

Overall, our device offers several potential improvements over existing microfluidic systems. First, our device allows rapid and easy connection of an electrical stimulation apparatus, simply requiring placement of the electrode in an input hole. Second, it can precisely modulate electrical stimulation, allowing graded stimulation of neural circuits with virtually any desired stimulation paradigm. Third, it is cost-effective. In particular, there are no added production costs associated with the stimulation feature because no microelectrode arrays or PDMS-embedded microelectrodes are needed. Finally, it is universally applicable to various types of neural circuits. Because our device is designed to include an axonal channel and dendritic channel systems with a synapse channel, it could easily be extended to study other neural circuit systems.

Our microfluidic device system will allow for intensive investigations into the mechanisms by which specific molecular players in synapses of CStr circuits modulate circuit functionality and could help identify the pathophysiological features of the CStr circuit associated with ASD and PD. It can also be extended to physiological studies of other neural circuits and related neurological diseases.

## Supplementary Information

**Additional file 1: Movie S1.** Live imaging of Ca^2+^ dynamics in response to 10 APs using Fluo5F-AM.

**Additional file 2: Movie S2.** Live imaging of Ca^2+^ dynamics in response to 10 APs using synaptophysin-GCaMP6f.

**Additional file 3: Movie S3.** Live imaging of vGlut-pHluorin fluorescence in response to 100 APs.

## Data Availability

There are no additional data. Additional video files are available online.
